# Effect of sequential electrospinning and co-electrospinning on morphological and fluid mechanical wall properties of polycaprolactone and bovine gelatin scaffolds, for potential use in small diameter vascular grafts

**DOI:** 10.1186/s40824-021-00240-8

**Published:** 2021-11-20

**Authors:** Yuliet Montoya, José Cardenas, John Bustamante, Raúl Valencia

**Affiliations:** 1 Grupo de Automática y Diseño A+D, Universidad Pontificia Bolivariana, Medellín, Colombia; 2 Grupo de Dinámica Cardiovascular, Centro de Bioingeniería, Universidad Pontificia Bolivariana, Medellín, Colombia; 3 Comité de Trabajo de Bioingeniería Cardiovascular, Sociedad Colombiana de Cardiología y Cirugía Cardiovascular, Bogotá, Colombia

**Keywords:** Small diameter vascular graft, Sequential and co-electrospinning, Morphology of scaffolds, Physicochemical properties, Fluid-mechanical properties

## Abstract

**Background:**

Nowadays, the engineering vascular grafts with a diameter less than 6 mm by means of electrospinning, is an attracted alternative technique to create different three-dimensional microenvironments with appropriate physicochemical properties to promote the nutrient transport and to enable the bioactivity, dynamic growth and differentiation of cells. Although the performance of a well-designed porous wall is key for these functional requirements maintaining the mechanical function, yet predicting the flow rate and cellular transport are still not widely understood and many questions remain open about new configurations of wall can be used for modifying the conventional electrospun samples. The aim of the present study was to evaluate the effect of fabrication techniques on scaffolds composed of bovine gelatin and polycaprolactone (PCL) developed by sequential electrospinning and co-electrospinning, on the morphology and fluid-mechanical properties of the porous wall.

**Methodology:**

For this purpose, small diameter tubular structures were manufactured and experimental tests were performed to characterize the crystallinity, morphology, wettability, permeability, degradability, and mechanical properties. Some samples were cross-linked with Glutaraldehyde (GA) to improve the stability of the gelatin fiber. In addition, it was analyzed how the characteristics of the scaffold favored the levels of cell adhesion and proliferation in an in vitro model of 3T3 fibroblasts in incubation periods of 24, 48 and 72 h.

**Results:**

It was found that in terms of the morphology of tubular scaffolds, the co-electrospun samples had a better alignment with higher values of fiber diameters and apparent pore area than the sequential samples. The static permeability was more significant in the sequential scaffolds and the hydrophilic was higher in the co-electrospun samples. Therefore, the gelatin mass losses were less in the co-electrospun samples, which promote cellular functions. In terms of mechanical properties, no significant differences were observed for different types of samples.

**Conclusion:**

This research concluded that the tubular scaffolds generated by sequential and co-electrospinning with modification in the microarchitecture could be used as a vascular graft, as they have better permeability and wettability, interconnected pores, and a circumferential tensile strength similar to native vessel compared to the commercial graft analyzed.

## Introduction

Peripheral vascular disorders are associated with blockages and obstructions of small diameter vessels (<6 mm) leading to reduced blood flow and tissue detriment due to poor nutrient supply, therefore, angioplasty is commonly performed with the use of stents or surgical bypass grafts [1–3]. Each year, cardiovascular diseases generate about 17.9 million deaths [4]. Additionally, the number of invasive vascular procedures in the lower extremities has doubled in the last decade. For this type of pathologies, grafts of biological origin are usually used, such as autologous (from the same patient) and homologous (from a donor of the same species) [5], [6]. However, they have clinical disadvantages in their application due to a lack of donors, anatomical variability, or patients with previous interventions [7], [8]. Another option for vascular replacement is the use of grafts made of non-biodegradable polymers such as Teflon® or Dacron®, which have been efficient in replacing large diameter vessels. However, when applied to the treatment of smaller vessels, they present complications related to thrombotic occlusion or intima hyperplasia due to their low biocompatibility [9].

In this sense, cardiovascular tissue engineering is key for the development of porous structures useful for replacing damaged tissues in the vascular system [10], [11], and for the evaluation of the biocompatibility of a variety of biomaterials [12], [10]. Biodegradable polymers of synthetic and natural origin [7], [11], are an alternative for the design of porous walls that provide a biological microenvironment that facilitates the development of new tissue while maintaining the mechanical function [12]. The study of natural polymers or proteins as electrospun scaffolds has gained great interest since they have the inherent ability to bind cells because they contain specific protein sequences (arginine, glycine and aspartic acid) [7], [8]. Therefore, synthetic and natural polymers can be combined to improve physical, chemical, and biological properties [13, 14]. In this way, they increase biocompatibility with the cellular microenvironment, favoring a tissue functional response [15], [16].

**Fig. 1:**
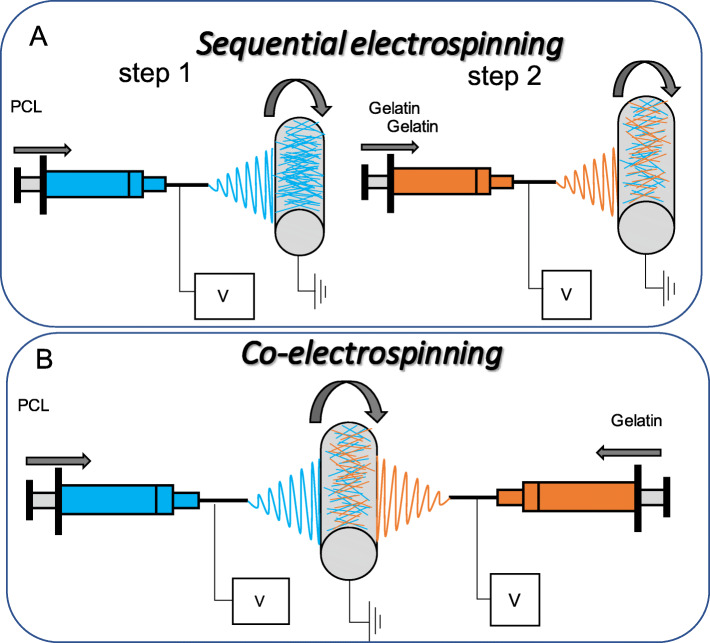
Schematic of sequential and co-electrospinning setup where the position of the needles can be next or opposite to each other: (**A**) sequential setup can be used to fabricate layer-by-layer structure; (**B**) co-electrospinning setup can be used to fabricate mixed fibrous structure

Electrospinning has been shown as an alternative method for the development of scaffolds with nano and micro-fibrous morphology, more similar to a native extracellular matrix [17]. With this technique, it is possible to design porous walls using different configurations that allow to vary the morphological characteristics such as the diameter of fibers, their orientation, and the size of the pores. The spatial organization of fibers in these structures is a significant parameter for the integrity of the scaffold, porosity, and cellular behavior [18]. Using conventional electrospinning, scaffolds with homogeneous characteristics in their thickness are obtained, however, the wall of a native vessel has variable characteristics, for which configurations as the sequential electrospinning and co-electrospinning are modifications of the conventional electrospinning technique, to develop scaffolds with multiple layers or variable morphologies to imitate a specific morphology of extracellular matrix [19]. Sequential and co-electrospinning setup allows the preparation of different structures (layer-by-layer or mixed fibrous) composed of different polymers, for example a natural polymer and a synthetic polymer. The position of the needles can be next or opposite to each other, as seen in Fig. 1.

Besides, the electrospinning parameters allow variation in porosity through the combination of modification of pore diameter and fiber deposition [18]. It has been found that endothelial cells tend to align in the microfiber scaffold and to allow the penetration through the pores, while in the nanofiber scaffolds, the cells have a random growth with a minimum ability to infiltrate [20]. Additionally, the orientation of fibers in one direction minimizes the space between them, reducing the pore size; while that the alignment can be manipulated using a rotating collector with variable-speed or controlling the electric field [21, 22]. It has been reported that aligned fibers help in the orientation and elongation of smooth muscle cells through the longitudinal axis [23].

Static permeability is another property that allows relating to the performance of a vascular graft. It has been reported that values of hydraulic conductivity lower than 600 mL/cm^2^/min limit the microcirculation of nutrients or biomolecules through the wall in hydrophobic grafts [24]. For scaffolds that exhibit hydrophilic behavior, this value may be lower since they allow to contain volumes of vascular fluid throughout its three-dimensional structure, which allows an adequate molecular transport (oxygen, electrolytes, glucose, etc.). In this way, polymers of natural origin such as proteins provide added value due to their hydrophilicity that allows an initial absorption of water [25].

Another challenge in the design of electrospun vascular grafts is to ensure adequate properties of mechanical strength and elasticity to mimic those of a blood vessel [26]. Differences in mechanical properties can cause damage to endothelial cells, hyperplasia, or ruptures in the device after implantation [27].

According to this context, the purpose of this study is to evaluate the effect of sequential electrospinning and co-electrospinning on crystallinity and morphological characteristics, permeability, wettability, degradability, and mechanical properties. Additionally, the morphological and fluid-mechanical properties were characterized in a commercial graft of 6 mm internal diameter and compared with the scaffolds developed in this research.

## Materials and methods

### Materials

PCL (Mw: 80,000) pellets and bovine gelatin type b (225 g bloom) were purchased from Sigma-Aldrich. N, N-dimethylformamide (DMF), dichloromethane (DCM), and acetic acid were purchased from Panreac. GA 25 % v/v for the crosslinking was purchased from Protokimica, CO. All the solvents were used without further purification. The NIH/3T3 fibroblast cell line was purchased from the American Type Culture Collection (ATCC), and Dulbecco’s Modified Eagle’s Medium (DMEM) and phosphate-buffered saline (PBS) were obtained from Lonza. Fetal bovine serum (FBS) was purchased from Microgen and hematoxylin - eosin was purchased from Thermo Fisher Scientific.

### Electrospinning

In this study, scaffolds were made using two types of electrospinning, in search of a microarchitecture for application in the vascular area. For sequential scaffolds, solutions of PCL 15 % (w/v) in DMF:DCM 1:1 and gelatin 25 % (w/v) in acetic acid 20 % (v/v) were loaded into 5 mL syringes with a 21 G cutoff tip. 6 syringes of each solution were mounted on an infusion pump (Cole Parmer 60,061), using PCL flows of 1.2 mL/h and 0.8 mL/h. These samples were labeled as B0 and C0 respectively. In both cases, the gelatin flow was 0.3 mL/h. Initially. First, a volume of 2.2 mL per syringe of PCL solution was dispensed, followed by 0.8 mL of gelatin solution per syringe. Co-electrospun scaffolds were made dispensing simultaneously with the PCL and gelatin solutions. These samples were labeled as B1 using a PCL flow of 1.2 mL/h and C1 for a 0.8 mL/h flow. In all the cases for the PCL, a voltage of 18 kV was applied to the solution using a power source (Gamma High Voltage Research 50PN) and a distance of 18 cm was set between the collector and the needles. A voltage of 15 kV and a distance of 15 cm was used for the gelatin. Samples were collected in a 6 mm diameter rod wrapped with aluminum foil and grounded, rotating at a speed of 20 rpm. For mechanical characterization, tubular samples were collected using a 6 mm diameter cylinder wrapped with aluminum according to [28]. The relative humidity in all cases was in a range between 45 % and 55 % and the temperature was close to 30 °C. All the operating parameters were tested in a previous standardization work based on the literature [23, 29–35]. Table 1 summarizes all the operation parameters.

**Table 1:** Summaries of electrospinning operation parameter

Scaffold name	B0		C0		B1		C1	
Polymer	PCL	Gelatin	PCL	Gelatin	PCL	Gelatin	PCL	Gelatin
Technique	sequential electrospinning				co-electrospinning			
Flow (mL/h)	1.2	0.3	0.8	0.3	1.2	0.3	0.8	0.3
Voltage (kV)	18	15	18	15	18	15	18	15
Needle-rod gap (cm)	18	15	18	15	18	15	18	15
Rod diameter (cm)	0.6							
Rod rotation speed (rpm)	20							
Humidity (%)	40-50							
Temperature (°C)	28-30							

### Crosslinking treatment

The scaffolds were cross-linked with GA based on [36–39]. For this, a Petri plate was disposed in the bottom of a desiccator with 0.8 mL of GA at 25 % v/v in aqueous solution (these conditions were tested in a design of experiments not reported in this work). At the top of the desiccator, the samples were placed on a ceramic mesh. The desiccator was closed with a vacuum for 48 h to allow cross-linking. This procedure is used to improve the stability of the gelatin fiber, in interaction with aqueous solvents, due to the union between the free amino groups of lysine or hydroxylysine residual amino acid present in the protein with the aldehydes of GA [39].

### FTIR

To evaluate the effect of crosslinking, sequential and co-electrospun scaffolds were analyzed to determine changes in functional groups of the natural and synthetic polymer without and with treatment. For this, an infrared Fourier transform spectrophotometer with ATR module was used (FTIR, Thermo Scientific iS50) at a resolution of 4 cm^− 1^ and 32 scans. Then, using the Origin Lab® software, the Fourier deconvolution of the infrared spectra covering the amide I region was performed (1600-1700 cm^− 1^).

### Morphological characterization

Samples were analyzed in a scanning electron microscope (SEM, JEOL JCM-6000 Plus) operating at 15 kV to determine the morphological changes in the scaffolds. For each type of electrospun, three micrographs were processed and analyzed using the Fiji software with the plugin DiameterJ [40–42]. From the analysis of the micrographs, the following parameters were determined: fiber diameter, orientation, and apparent pore area. the segmentation algorithm applied to each micrography was based on the methodology described in [41].

### Wettability

The wettability of the membranes was measured by contact angle (Contact Angle System OCA Dataphysics®) based on ASTM D7334-08 [43], to determine the hydrophilicity of scaffolds. For this test, a syringe dispenser was loaded with simulated body fluid (SBF) fabricated according to [44]. Subsequently, a drop of 10 µL of the solution was deposited on the sample, and photograms were captured for 30 s. For each scaffold, a sample with dimensions 2 cm x 5 cm was cut out and 4 drops were dispensed over this. For this analysis, three replicates were used for each type of sample.

### Static permeability

For the measurement of static permeability, the method stipulated in ISO7198 was adapted [45] with which the resistance to the passage of the flow through the microarchitecture of the scaffold wall was determined. The test bench consisted of a column of hydrostatic pressure, with a height of liquid equivalent to physiological, hypotensive and hypertensive pressures (0 to 150 mmHg). The test consisted of the measure of distilled water mass passing through the scaffolds by 1 min, the scaffolds were arranged in a sample holder with a 1.1 cm of hole diameter. The evaluated pressures were 50, 80, 120, and 150 mmHg. To ensure the reproducibility of the results, two replicates were analyzed for each type of membrane. With the results obtained, the permeability was calculated using (1):

1 $$K=\frac{{m}_{fluid}}{{\rho }_{fluid} \times {A}_{hole}\times t}$$

where: ${m}_{fluid}$ distilled water mass [g], ${A}_{hole}$ hole area [cm^2^], $t$ time [s], $K$ permeability [mL/min/cm^2^] and ${\rho }_{fluid}$ fluid density [g/mL].

### Degradation

The degradation rate of the membranes was evaluated in media with neutral pH to determine the loss of biomaterial in interaction with fluids. For the test, tubular segments with and without cross-linking treatment were used, with dimensions of 1.5 cm long by 6 mm in diameter. Then, the samples were immersed in phosphate-buffered saline solution (PBS) 1X during a time of 24, 48, 72, and 240 h to measure the weight loss due to degradation [46]. To obtain reproducibility and repeatability, three replicates were analyzed for each type of scaffold.

### Mechanical characterization

The purpose of the test was to evaluate the circumferential tensile strength and creep elongation of bilayer membranes based on an adaptation of the standard ASTM D638 [47]. For the test 2 copper fasteners were used. Three tubular samples of each type of electrospun scaffold were cut with 2 cm length to be tested in a universal testing machine (Instron ® 5582) with a load cell of 1 kN with a speed of 10 mm/min. To ensure the reproducibility of the results, three replicates were analyzed for each type of membrane.

### Cell adhesion and proliferation an *in vitro* model of 3T3 fibroblasts

Cultures of 3T3 fibroblasts were maintained in DMEM media supplemented with 10 % FBS, penicillin (100 U/mL), and streptomycin (100 µg/mL). The culture medium was changed every three days, and the cell culture was maintained at 37 °C, 5 % CO_2_, 95 % O_2_, and 95 % relative humidity. Before the interaction of the membranes with the *in vitro* model of 3T3 fibroblasts, these were sterilized and preconditioned with DMEM without supplementation for 24 h at 37 °C. For the cell adhesion and proliferation assay, 30 × 10^3^ cells/well of 3T3 fibroblast, with viability greater than 90 %, were seeded on the cross-linked scaffolds for 24, 48, and 72 h. After each incubation period, the scaffolds were removed and fixed with 10 % formaldehyde for 30 min. Over time, it was added with hematoxylin/eosin to stain plasma membranes and cell nuclei. Finally, frames were obtained at 10 X magnification using the Optika Vision Pro software, and cell proliferation was determined from image processing with ImageJ® software. Before the interaction of the electrospun scaffolds with the *in vitro* model of 3T3 fibroblasts, these were sterilized with UV radiation and preconditioned with DMEM without supplementation for 24 h at 37 °C.

### Statistical analysis

The results obtained in the characterizations were statistically analyzed by analysis of variance, to determine the influence of each of the factors and their relationship with the fluid-mechanical properties. This was done using Statgraphics software. Data are presented as mean ± standard deviation and a statistical significance of 0.05 was used.

## Results

### FTIR

**Fig. 2:**
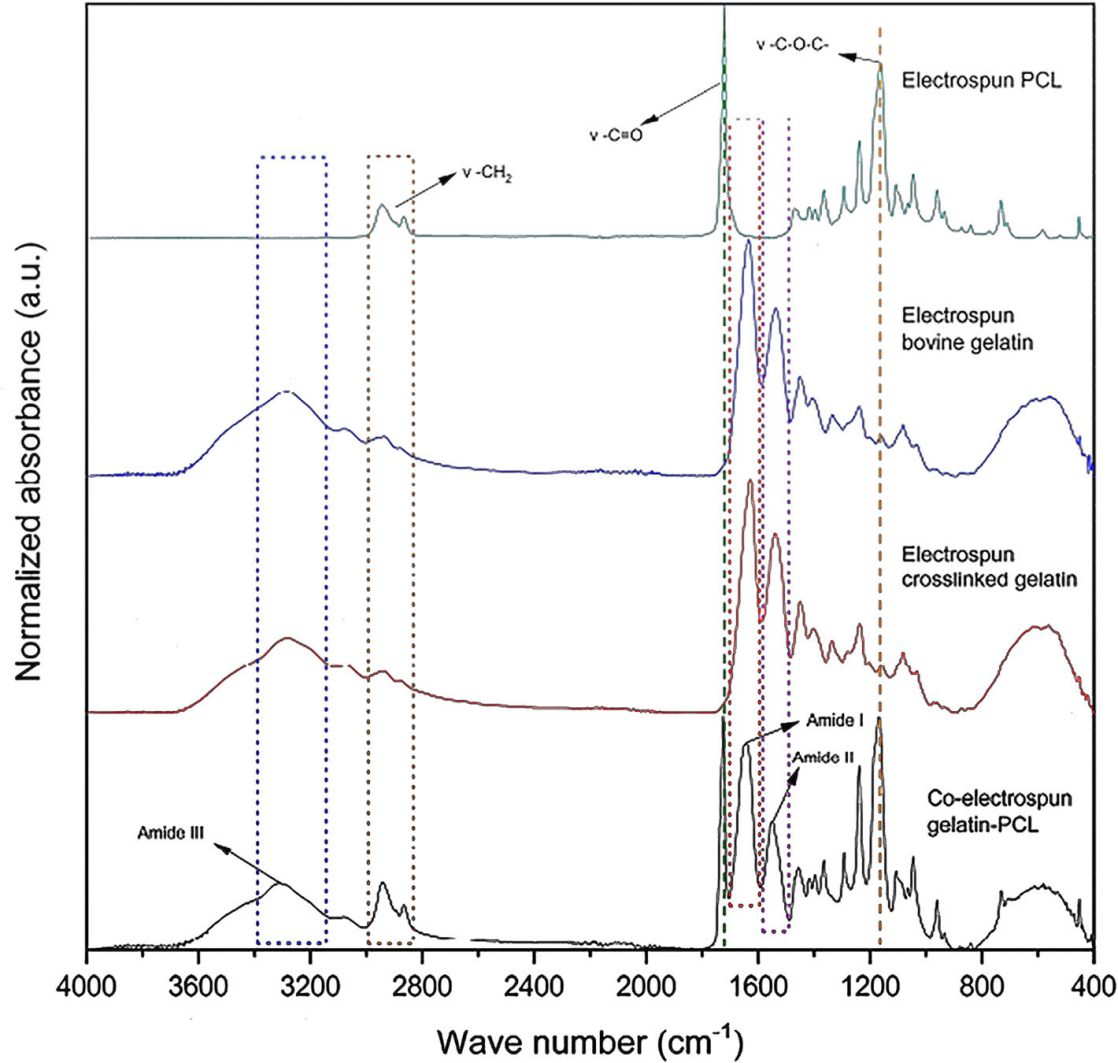
Normalized FTIR spectra of sequential electrospun membranes of the gelatin layer without and with crosslinking. A representative spectrum of sequential electrospun membranes in the PCL layer. A representative spectrum of co-electrospun membranes

Figure 2 shows the normalized absorbance spectrum IR with the corrected baseline for the gelatin layer of the sequential scaffolds with and without crosslinking and for a co-electrospun scaffold to flows of 0.8 and 1.2 mL/h. The FTIR analysis shows the presence of the amide bands I, II, and III which are common structures in bovine gelatin and stretches of groups of CH_2_, C=O, and C-O-C from PCL groups, as evidenced in Table 2. Due to the presence of all of these stretches in the co-electrospun scaffold, the generation of a biocomposite material is determined. On the other hand, in the amide I, structures related to the stretching of the -C=N- bond stretch are generated in the gelatin crosslinking with GA [48]. In addition, it is observed that the spectra of the sequences electrospun membranes show characteristic bands of the synthetic polymer (PCL) and the protein (gelatin) in two different layers. While the co-electrospun membranes show characteristic bands of both polymers in the same spectrum. Which is related to the coexistence of PCL / Gel. These characteristics are related to the type of technique used to manufacture the membranes.

**Table 2:** Vibration range of groups present in scaffolds

Material	Wave number range (cm^−1^)	Associated band	Scaffold present in
**PCL**	2991-2901	Asymmetric stretch CH_2_	PCL layer sequential, co-electrospun
	2879-2840	Symmetric stretch CH_2_	PCL layer sequential, co-electrospun
	1753-1691	Stretch C=O	PCL layer sequential, co-electrospun
	1208-1127	Asymmetric stretch C-O-C	PCL layer sequential, co-electrospun
**Gelatin**	1702-1596	Amide I (Stretch C=O)	Gelatin layer sequential, crosslinked gelatin layer sequential, co-electrospun
	1588-1495	Amide II (Vibration, bending N-H and stretch, vibration C-N)	Gelatin layer sequential, crosslinked gelatin layer sequential, co-electrospun
	3374-3147	Amide III (Stretch, vibration C-N and N-H)	Gelatin layer sequential, crosslinked gelatin layer sequential, co-electrospun

### Morphological characterization

**Fig. 3:**
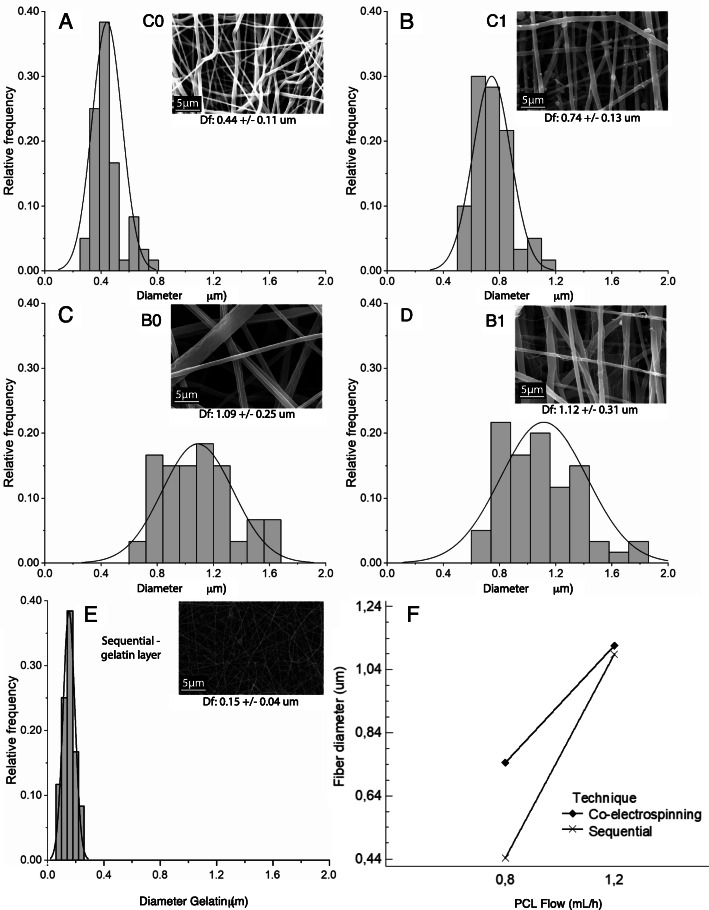
Histograms of the distribution of fiber diameter: (**A**) Interaction graph according to the technique) sample with a flow of 0.8 mL/h in the sequential configuration; (**B**) sample with a flow of 0.8 mL/h in the co-electrospinning configuration; (**C**) sample with a flow of 1.2 mL/h in the sequential configuration; (**D**) sample with a flow of 1.2 mL/h in the co-electrospinning configuration; (**E**) gelatin layer in the sequential configuration; (**F**) Interaction graph between technique and flow

The results of average fiber diameter and the distribution in the micrographs are shown in Fig. 3. The histograms of the samples B0 and B1 fabricated with a flow of 1.2 mL/h (Fig. 3 C and Fig. 3D) had higher diameters (1.09 and 1.12 μm), although with a wider variability compared to C0 and C1 at a flow of 0.8 mL/h (0.44 and 0.74 μm) (Fig. 3 A and Fig. 3B). These results agree with [12, 15, 17], where the fiber diameter is directly proportional to the solution flow. Additionally, a network of fibers with a smaller diameter around 0.15 ± 0.041 μm corresponding to the gelatin is presented in the co-electrospun scaffolds. The average diameter of PCL fibers in co-electrospun scaffolds is greater than that of sequential scaffolds, possibly since the opposition of fields reduces the intensity of the voltage, therefore a lower level of tightening of fibers is obtained [49].

Due to the variation coefficient for the fiber diameter was greater than 20 % for B0 and C0 in the PCL layer, and B1, Mood’s median test were performed for the PCL flow and the electrospinning technique [50]. Since a P-value for the Chi-square test of 0.020 was lesser than 0.05, it can be affirmed that the fiber diameters obtained after varying the technique and PCL flow have a statistically significant difference with a level of confidence of 95 % (Fig. 3 F).

**Fig. 4:**
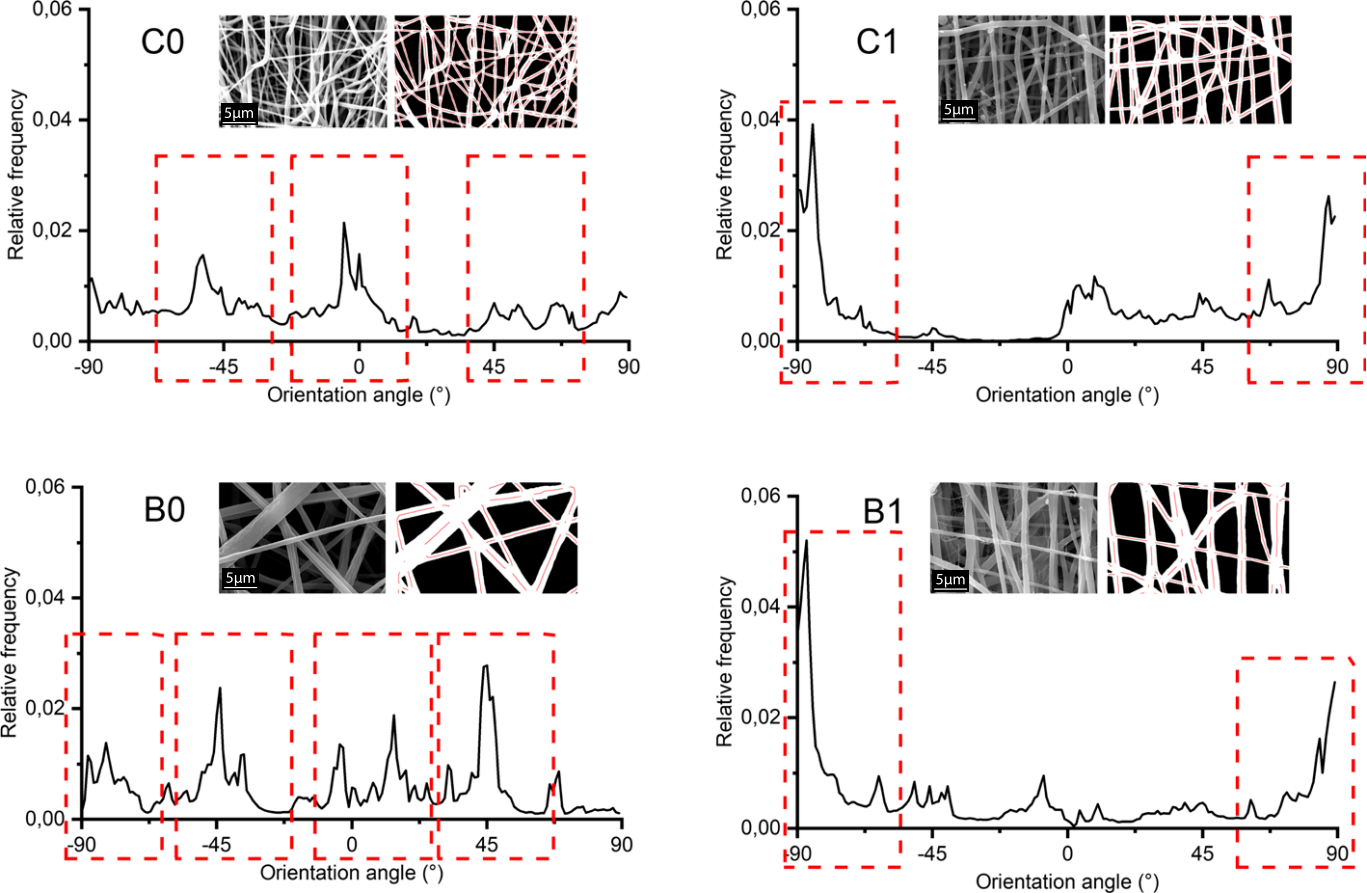
Normalized global frequency graphs of the fiber orientation. The fiber of sequential scaffolds shows multiple peaks of relative frequency, while in co-electrospun scaffolds predominant peaks are observed around 90 °, indicating a greater alignment. Where C0 is a sequential membrane and C1 is a co-electrospun membrane at 0.8 mL / h and B0 is a sequential membrane and B1 co-electrospun membrane at 1.2 mL / h

**Fig. 5:**
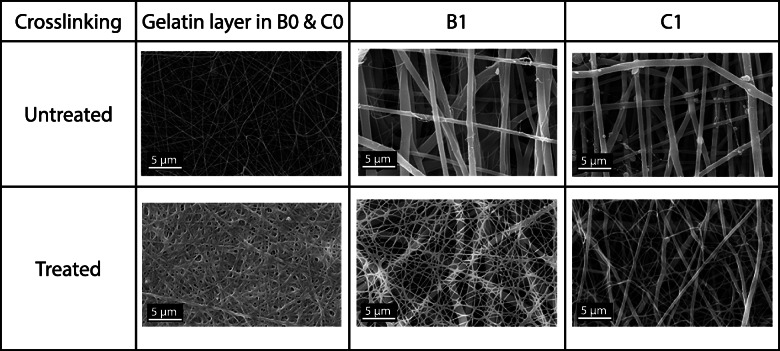
Comparison of SEM micrographs of the electrospun scaffold, before and after the crosslinking treatment

The cells and fibers of the extracellular matrix in most natural tissues exhibit well-defined patterns and specific spatial orientations [51]. Additionally, it has been reported that cell adhesion and proliferation is significantly improved in scaffolds with aligned morphologies since they allow to guide cell growth along the fibers. In order to analyze this behavior, the normalized frequency graphs for the fiber orientation angle were obtained in Fig. 4. It was found that the sequentially fabricated scaffolds generate fibers with random directions due to multiple frequency peaks at different angle values were observed. Unlike the co-electrospun scaffolds, there is a greater tendency to organize the fibers in the vertical direction since a predominant peak at -90 ° and 90 ° is observed in both flow conditions. Because of the methodology used to determine fiber diameters is difficult to determine the diameter of the small gelatin fibers present in the co-electrospun scaffolds, an apparent pore area analysis was performed, which gives valuable information about the processes of cellular infiltration to generate pseudo-endothelization of the graft. Figure 5 shows the micrographs for each type of scaffold and it can be seen that the fibrous structure of the gelatin layer of the sequential samples B0 and C0 is preserved.

Table 3 shows the average apparent pore area measurement of electrospun samples without and with treatment. It is found that the apparent pore area is reduced due to the cross-linking treatment of about 60 % and 49 % in B1 and C1 samples respectively. Despite this, the B1 scaffolds have an apparent pore area 46 % greater than C1 after treatment. On the gelatin layer in the sequential scaffolds, it was not possible to determine the apparent pore area after treatment with the image processing software due to swelling and bonds in the fibrous network.

**Table 3:** Average apparent pore area of electrospun scaffolds

Average apparent pore area			
Scaffold	Untreated (µm^2^)	Cross-linked (µm^2^)	Reduction percentage (%)
B0-C0 gelatin layer	0.22 ± 0.28	--	--
B1	13.30 ± 11.88	5.31 ± 3.53	60.07
C1	7.27 ± 5.29	3.64 ± 1.56	50.00

The results show that varying from a flow of 0.8 to 1.2 mL/h, the diameter of the fibers increases a greater percentage in the sequential configuration (145.6 %) compared to the co-electrospinning (49.8 %). Working conditions leads to the flow has a greater effect on the diameter of the fibers when they are fabricated using the sequential configuration.

### Wettability

**Fig. 6:**
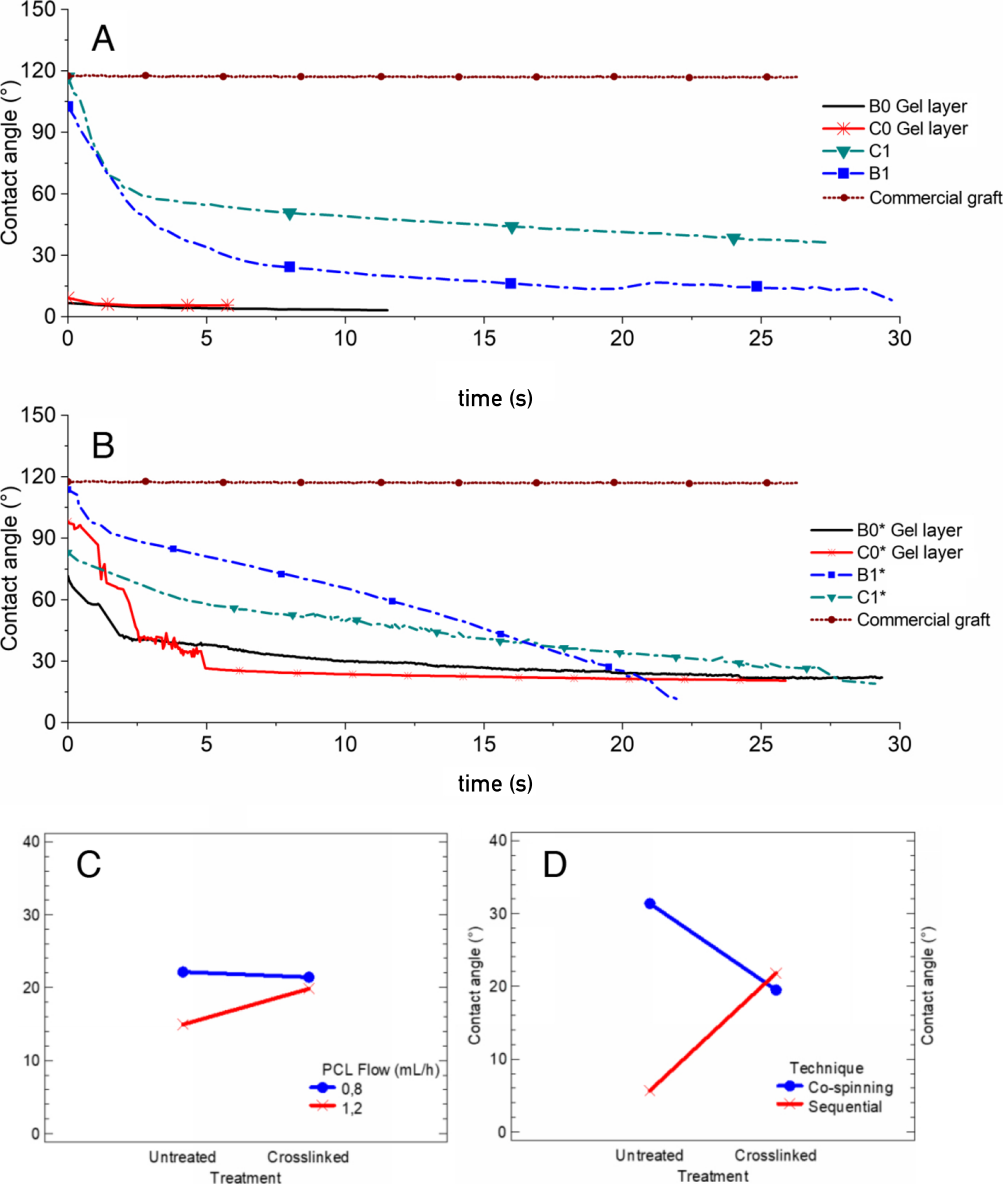
Contact angle vs. time curves: (**A**) Electrospun scaffolds without crosslinking; (**B**) Crosslinked electrospun scaffolds; (**C**) Interaction graph according to the PCL flow; (**D**) Interaction graph according to the technique

Figure 6 A and Fig. 6B show the contact angle as a function of time for untreated and crosslinked scaffolds respectively. For the gelatin layer in the scaffolds obtained by sequential electrospinning without treatment presented a contact angle lower than 10° and was only detectable by the measuring software for 12 s before the drop was completely absorbed. Additionally, during the characterization of the gelatin layer wettability in the sequential samples, it was found that this protein dissolved upon contact with the drop of simulated body fluid which evidenced a low resistance in aqueous environments when they did not have the treatment. In contrast, when applying the cross-linking process, the contact angle was initially around 80° and slowly decreased to stabilize with a value of about 20° after 5 s, evidencing a greater resistance to aqueous environments of the protein possibly due to the crystallinity level increased.

Additionally, with the analysis of variance according to the contact angle, it was found that the electrospinning technique affected with statistical significance because its P value was 0.025 lesser than the test statistic 0.05.

For the commercial graft (Fig. 6 A and Fig. 6B), a contact angle value of about 120° was obtained and remained constant during the test. This is because the material of this graft is composed of a hydrophobic polymer (polytetrafluoroethylene) and possibly in its wall, the level of cell adhesion would be lesser because it is not less than 75° as the literature suggests [52].

According to the results obtained, crosslinking increased the value of the contact angle in the gelatin layer in the sequential scaffolds by about 283 % as seen in Fig. 6 C and Fig. 6D, while in the co-electrospun scaffolds the contact angle value after the treatment decreased by about 61 % compared to the un-crosslinked samples. On the other hand, when using a PCL flow of 1.2 mL/h the crosslinking increased the contact angle value by 33 % while using a flow of 0.8 mL/h the change due to the crosslinking was 3 %.

### Static permeability

**Fig. 7:**
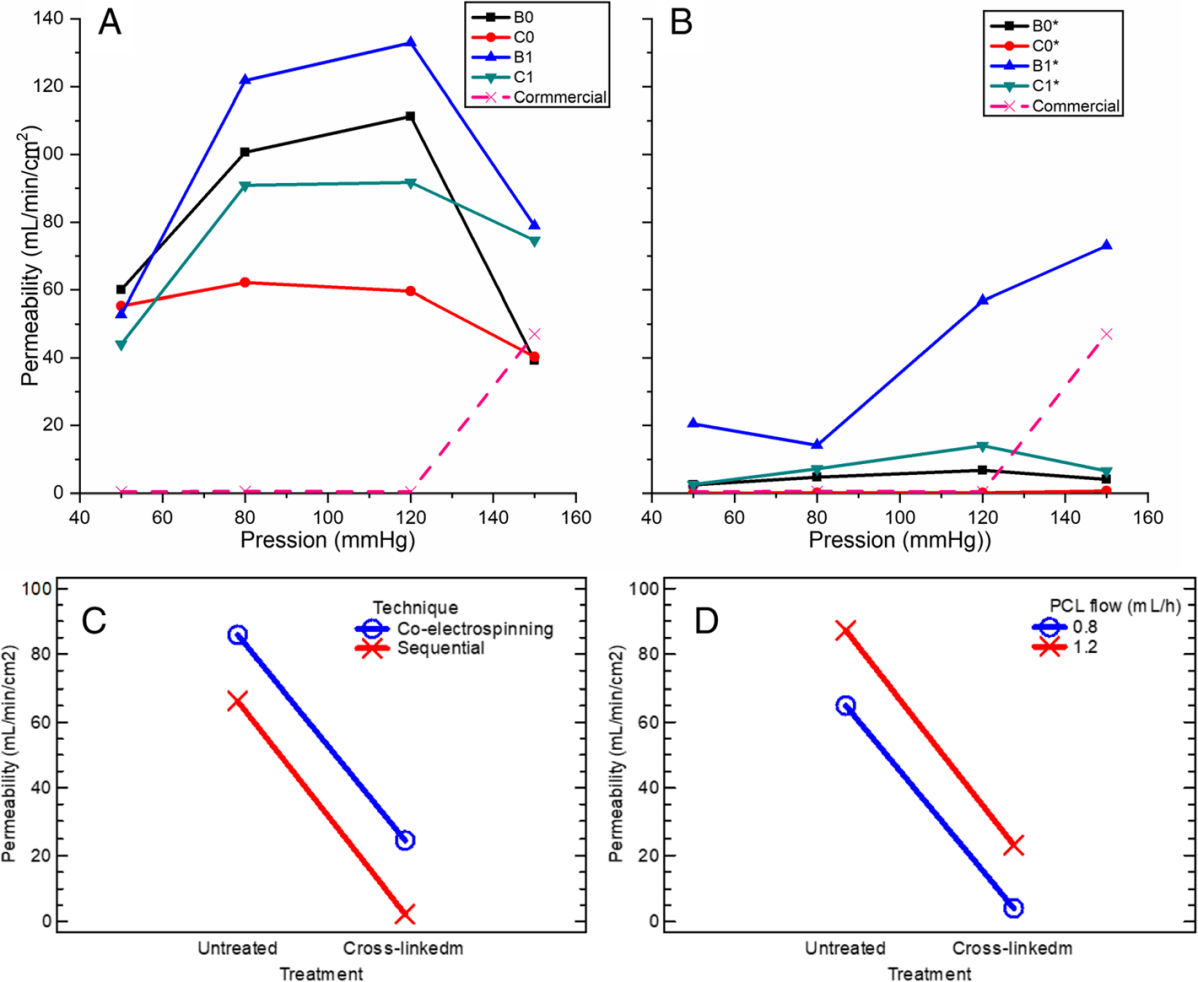
Static permeability: (**A**) Electrospun scaffolds without crosslinking; (**B**) Crosslinked electrospun scaffold; (**C**) Interaction graph according to the technique; (**D**) Interaction graph according to the PCL flow (mL / h)

Figure 7 A and Fig. 7B show the static permeability test as a function of hydrostatic pressure in a range of physiological and pathophysiological values for untreated and treated scaffolds respectively. These show that untreated scaffolds have a permeability in a range between 40 and 140 mL/min/cm^2^; while after the crosslinking treatment, permeability falls in a range between 0 and 20 mL/min/cm^2^ for sequential scaffolds B0, C0, and co-electrospun C1, while the B1 scaffold has a tendency to increase and reaches a permeability of up to 70 mL/min/cm^2^ at a pressure of 150 mmHg. On the other hand, the commercial graft permeability was zero, except at 150 mmHg where it had a permeability close to 50 mL/min/cm^2^. With an analysis of variance (ANOVA) for the static permeability, it was found that all the main effects had a statistical significance because their P-value was lesser of 1 × 10^−4^ for the technique, PCL flow, water pressure, and treatment and was lower than the test statistic 0.05.

Furthermore, the effect of crosslinking according to the electrospinning technique is shown in Fig. 7 C, where an average decrease in permeability around 72 % is shown for co-electrospun scaffolds and 96 % for sequential ones. The crosslinking had a greater effect on the sequential scaffolds because the gelatin layer formed a gel that when in contact with the water that sealed the structure and prevented the flow of water. On the other hand, for the co-electrospun scaffolds, the gelatin fibers were not agglomerated in a single layer but distributed throughout the three-dimensional structure of the wall, avoiding the generation of an occlusion. The reduction in permeability was possibly caused by the decrease in the average apparent pore area in the scaffolds. This same behavior is observed in Fig. 7D according to the PCL flow, where at a flow rate of 0.8 mL/h the permeability decreased an average of 94 %, while at a flow rate of 1.2 mL/h the decrease was 74 %.

The micrographs of the commercial graft show a discontinuous framework, which is interrupted throughout the wall structure avoiding interconnectivity between pores. Possibly due to this, in the static permeability tests values of 0 mL/min/cm^2^ were obtained at pressures of 50, 80, and 120 mmHg, since it does not allow the flow of the fluid through the wall. This type of graft probably does not favor cell infiltration or nutrients exchange through its wall, because according to the literature [24], vascular grafts with hydrophobic behavior must have permeability values greater than 600 mL/min/cm^2^ to ensure microcirculation of nutrients or biomolecules through their wall.

### Degradation

**Fig. 8:**
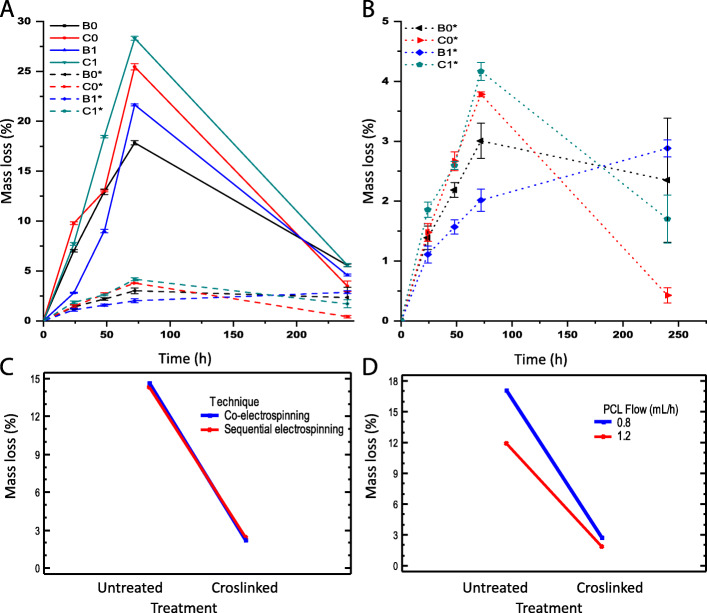
Mass loss of electrospun scaffolds: (**A**) samples without treatment; (**B**) cross-linked samples; (**C**) Interaction graph according to the technique; (**D**) Interaction graph according to the PCL flow (mL / h)

The loss of mass for scaffolds without GA treatment is shown in Fig. 8 A. It is observed that the scaffolds lose about 6.8 % ± 2.9 % of mass on average at 24 h, but B1 losses the least mass with a value close to 3 %. This could be because the gelatin meshed with the PCL fibers increases the tortuosity of the wall, which delay the dissolution of the gelatin compared to the sequential scaffolds, where the gelatin layer is completely exposed to the fluid.

After 48 and 72 h of the test, the average mass loss increased to 13.3 % ± 3.8 % and 23 % ± 4.55 % respectively. In this range of time, the C1 sample is the one that presents a greater loss of mass close to 19 % and 28 % respectively. A possible cause for this behavior is that this scaffold had an initial gelatin/PCL ratio of 3.8:6.2 higher than the ratio of 2.9:7.1 for B0, C0, and B1 samples. After an evaluation period of 240 h, it was found that electrospun membranes without crosslinking show an average loss of mass of 4.8 % ± 0.69 %, indicating degradation stability of electrospun membranes with low mass loss percentages. Which would favor tissue regeneration processes at the vascular level.

On the other hand, Fig. 8B shows the average loss of mass for the crosslinked scaffolds close to 3.2 % after 72 h of testing. Again, it is observed that C1 samples had the greatest weight loss during the test, which may be due to the greater ratio of gelatin/PCL in this scaffold. In contrast, B1 had a lesser weight loss at all evaluated times, where possibly the tortuosity of the scaffolds protects the gelatin inside the wall. After 240 h, the reticulated electrospun membranes presented an average loss of mass of 1.8 % ± 1.28 %, which represents that the samples exhibit mass losses of less than 3 %, which could favor processes of pseudoendothelialization and native tissue biointegration.

Additionally, an analysis of variance (ANOVA) for the mass loss was performed. It was found that the main effects PCL flow, crosslinking treatment, and immersion time had a statistical significance because their P-value was about 1 × 10^−4^ and lesser than the test statistic 0.05. It should be noted that the value of the F-ratio in the effects of treatment and time were greater than the others, so these have a greater statistical weight.

Figure 8 C shows the effect of crosslinking by the PCL flow, where there is evidence of a reduction in the average mass loss close to 14.4 % for a flow of 0.8 mL/h and 10.0 % for a flow of 1.2 mL/h. On the other hand, the interaction graph for crosslinking by the electrospinning technique is shown in Fig. 8D.

In the analysis of variance (ANOVA), this interaction did not present statistically significant importance, so it is observed that the sequential and co-electrospun curves overlap, and the average loss of mass is reduced by about 12.2 % due to crosslinking.

### Mechanical characterization

**Fig. 9:**
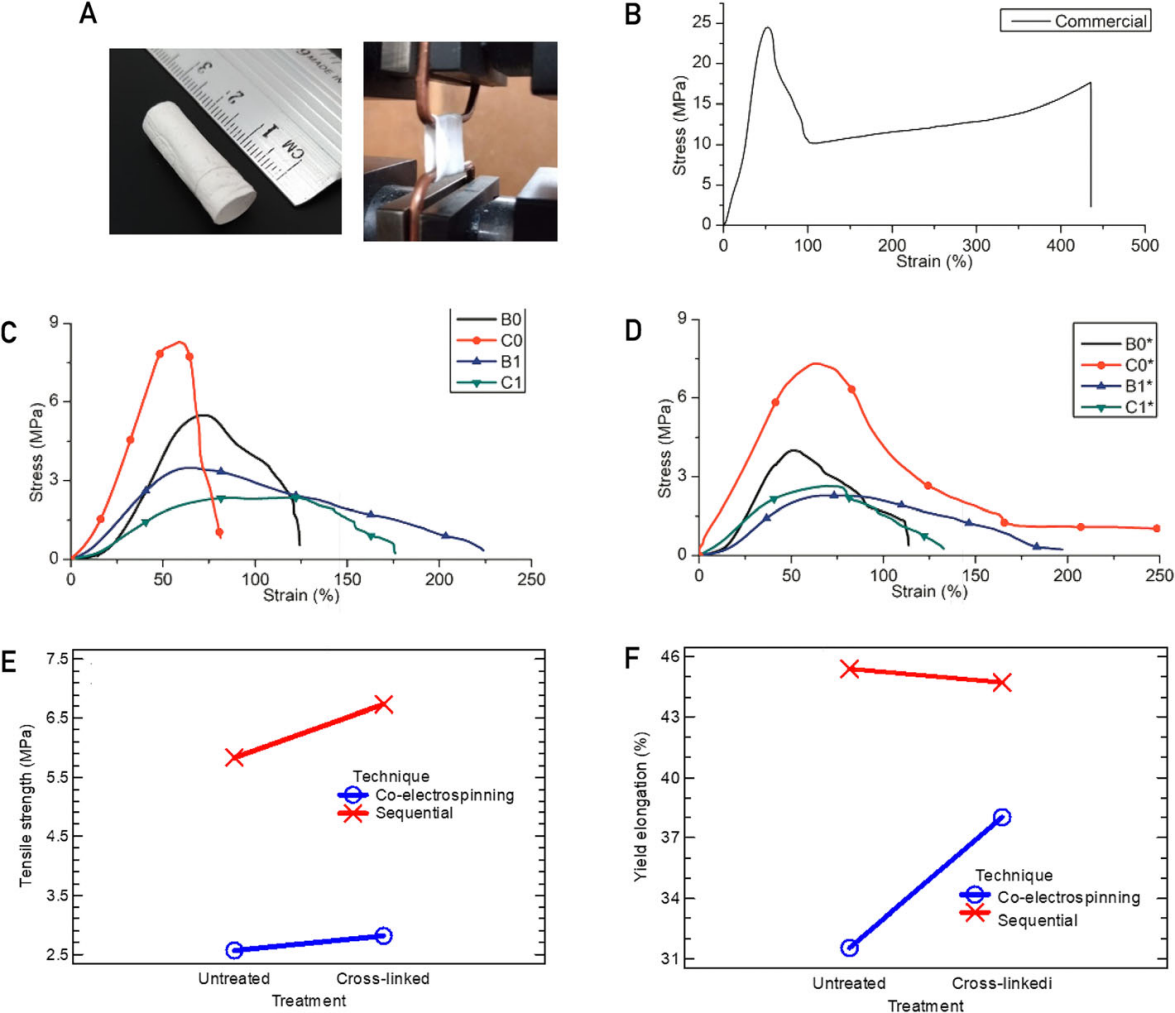
Stress curves vs. deformation for tubular scaffolds: (**A**) test of traction; (**B**) commercial sample; (**C**) sample without treatment; (**D**) sample with crosslinking treatment; (**E**) Interaction graph for tensile strength according to the technique; (**F**) Interaction graph for yield elongation according to the technique

Figure 9 C and Fig. 9D show the stress vs. strain curves for each type of scaffold in the circumferential direction, both untreated and crosslinked respectively. It is observed in both figures that the C0 samples are the scaffolds with greater tensile strength, although, after the cross-linking process, its tensile strength decreased by 15 % while its yield elongation presented a change of 2.4 %, a value without statistical significance. On the other hand, the C0 samples are the ones with the highest tensile strength because they have the smallest average fiber diameter.

To analyze the mechanical resistance data statistically, a variance test is performed with the data in Table 4, where a summary of the mechanical properties of yield elongation and tensile strength of tubular scaffolds are shown. With the Anova for the tensile strength, it was found that the technique and the flow are the effects with statistical significance because they have a P-value was 1 × 10^−5^ and 0.016 respectively which are lesser than the test statistic of 0.05. And an ANOVA test for the elongation was also applied and it was found that the electrospinning technique was the factor that had statistical significance with a P-value of 0.028 lesser than the test statistic of 0.05.

**Table 4:** Summary of mechanical properties of electrospun tubular scaffolds

	Yield strain (%)	Tensile strength (MPa)
**Untreated**		
B0	44.59 ± 13.78	5.09 ± 0.56
C0	44.88 ± 0.088	8.37 ± 0.12
B1	38.72 ± 3.23	3.46 ± 0.088
C1	37.37 ± 3.72	2.20 ± 0.22
**Treated**		
B0	46.91 ± 13.97	4.60 ± 0.84
C0	43.80 ± 1.45	7.08 ± 0.35
B1	29.54 ± 9.88	2.28 ± 0.006
C1	33.55 ± 4.88	2.87 ± 0.31

Figure 9E shows the combined effects for tensile strength of factors between Treatment-Technique. It is found that tensile strength only decreased by 13 % and 8 % for sequential and co-electrospun scaffolds respectively, due to crosslinking.

Figure 9 F shows the combined effects of yield elongation with the PCL flow-treatment. It was found that after the cross-linking procedure, ultimate elongation only increased 1 % for sequential scaffolds and decreased 17 % for co-electrospun scaffolds.

As in the previous case of tensile strength, the effect of crosslinking is not statistically significant due to the lower proportion of gelatin in the scaffold compared to PCL. Finally, a tensile strength value was obtained close to 25 MPa for the commercial graft (Fig. 9B) ) and it has been reported that native vessels with diameters close to 6 mm have circumferential tensile strengths between 3 and 13 MPa [26].

### Cell adhesion and proliferation

**Fig. 10:**
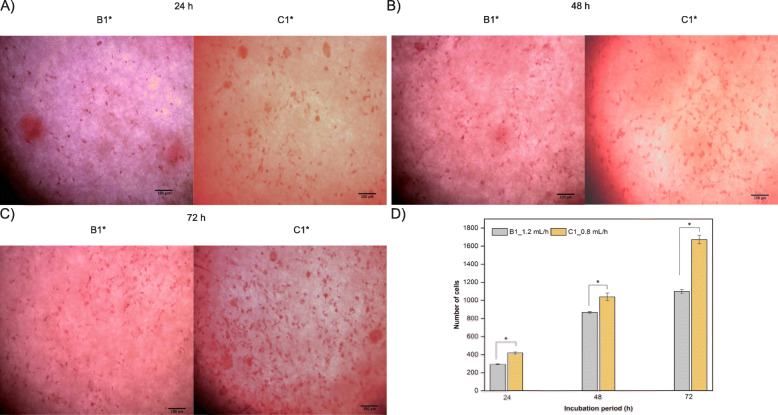
Cross-linked B1 and C1 membranes interacting with 3T3 fibroblasts: (**A-C**) bright-field images of fibroblasts; (**D**) cell growth rate at incubation periods of 24, 48, and 72 h

Proliferation characteristics were determined at incubation periods of 24, 48, and 72 h of interaction between the cross-linked membranes and 3T3 fibroblasts. Figure 10 A-C shows the hematoxylin-eosin staining images, where it can be seen that 3T3 fibroblasts adhered to B1 and C1. This was due to the physicochemical and morphological conditions of the membranes where the incorporation of a protein with the presence of the amino acid sequence arginine-glycine-aspartic acid promotes cell adhesion since this sequence is recognized by integrins, that promote the union of cells with the extracellular matrix [53]. On the other hand, it was found that membrane C1 at incubation periods of 24, 48, and 72 h exhibits a cell growth rate of 30 %, 17 %, and 52 % higher than B1 (Fig. 10D), respectively.

## Discussion

Efforts to obtain an adequate bioactivity, tissue engineering has promoted the use of polymeric structures of natural origin, which exhibit amorphous and crystalline structures. Derived from this, the use of bovine gelatin for the development of tubular structures, plays a major role in biocompatibility and pseudo-endothelialization processes. From the results, it is determined that deconvolution of the amide I band is more sensitive to changes in the content of secondary structures (beta turns and sheets) caused by crosslinking treatment. After the deconvolution, the area under the curve was obtained for the adjusted Gaussians around 1629, 1659, and 1693 cm^−1^, which represent the crystalline structures according to [29]. Furthermore, the percentage of these structures in the gelatin layer in sequentially electrospun scaffolds was found to around 49.8 %, while the sequential and co-electrospinning membranes exhibited an increase in the percentage of these crystalline structures after the crosslinking process with values of 55.9 % and 66.2 % respectively. Thus, it was found that, for the sequential scaffold with a crosslinked gelatin layer, the crystalline structures increased around 6 % compared to the untreated scaffolds; meanwhile for the crosslinked co-electrospinning scaffolds, the increase was around 16 %.

Due to the co-electrospun scaffolds had a lesser amount of gelatin on the surface, compared to the layer of this protein in the sequential scaffolds, the co-electrospun scaffolds reach a higher level of crystallinity at the level on the surface, when reacting with the same amount of GA. In addition, the gelatin in the co-electrospun scaffolds possibly may take a longer time to degrade compared to the sequential scaffolds.

On the other hand, tubular structures exhibit a microstructural reorganization thanks to the interrelation of polycaprolactone with gelatin, trying to emulate the biomimetics of a native vessel. From the morphological point of view, although in [21] the fiber orientation was changed by varying the rotation speed of the collector mandrel, it can be seen that the fabrication technique could also affect this morphological characteristic. The effect of electric fields in the alignment of the fibers in electrospun scaffolds can be evidenced in [22], where they modified the ground connection of the collector to obtain aligned fibers. According to these results, due to the increase in the alignment of the fibers in co-electrospun scaffolds, the tensile strength could be favored [54], because the maximum load in the direction of the fibers is increased.

Additionally, due to the presence of moisture during GA treatment, the morphology of the fibers is affected, causing the fibers to bond at the points of attachment. Also, crosslinking involves the reaction of free amino groups present on amino acid residues of polypeptide chains with the aldehyde group of GA, which generates a closeness of the fibers and their swelling, causing a significant reduction in the apparent pore area. On the other hand, in the co-electrospun scaffolds B1 and C1, the gelatin fibers are distributed among the PCL fibers, which reduces the approach between gelatin fibers and a greater apparent pore area.

Because in the sequential scaffolds there is an occlusion with almost zero pore area values on the gelatin layer due to chemical treatment, cell adhesion and proliferation could be lower and only at a superficial level, limiting wall penetration. While in the co-electrospun scaffolds B1 and C1 that have a larger pore area, they could enhance cell growth not only at the surface level but also inside the wall due to gelatin fibers are distributed throughout the thickness [55]. On the other hand, the presence of gelatin in these electrospun scaffolds possibly favors cell deposition as it contains amino acid sequences that work like attachment points for cells [14]. Additionally, due to the crosslinking treatment, the dissolution of the gelatin inside the scaffold would be avoided on the initial days where the adhesion and cell proliferation are developed [39].

Wettability is an important factor for scaffold performance in tissue engineering applications since hydrophilic surfaces with contact angles below 75° have been found to improve cell adhesion to the wall [52], [56]. For the PCL layer in the sequential scaffolds, contact angles greater than 110° were obtained, which coincides with the literature due to the nature of the apolar phase [57]. This characteristic was improved with the incorporation of bovine gelatin in the polymer mixture.

On the other hand, the contact angle of the co-electrospun scaffolds without cross-linking treatment tended to decrease from 110° to a stable value between 30° and 40° after 10 s. This behavior is probably due to both the gelatin and the PCL are present throughout the scaffold wall and allow the absorption of the fluid to be slower because the gelatin fibers are not fully exposed to the simulated body fluid. Additionally, it is observed that B1 reaches a lower value than C1 possibly because its apparent pore area is greater and would facilitate the absorption of the fluid. The decrease in contact angle value for crosslinked co-electrospun scaffolds reaches a stabilization close to 20 ° after 15 s. Similar results were reported in [23], [33] y [38], where contact angles between 23 – 52° were obtained for scaffolds fabricated with gelatin and a synthetic polymer like PCL. Moreover, the time required to stabilize the contact angle value of crosslinked scaffolds is longer compared to untreated scaffolds, which indicates a lower degradation of the protein in aqueous media [58].

Following this further, it has been reported that to ensure adequate cell infiltration and exchange of nutrients or biomolecules between cells in vascular graft, interconnected porosities are required [35], [59], which allows controlled permeability. It has been found that a hydrophilic wall allows containing a volume of vascular fluid between its structure, which makes possible an adequate molecular transport [24].

In the co-electrospun scaffolds, the presence of gelatin and after the cross-linking treatment, it is possible to retain a volume of water inside the wall which would allow continuous molecular transport by diffusion. Additionally, because gelatin has a rapid degradation in aqueous environments, the apparent pore area of these scaffolds would increase after a short period and possibly would facilitate cell proliferation [14]. In sequential scaffolds with cross-linking treatment, the occlusion that occurs on the gelatin layer could avoid the need to perform the pre-coagulation process and allow retaining vascular fluid in the wall [24].

Following this further, a mass loss test was performed on tubular scaffolds, to determine the effect of the cross-linking process on the time in which the gelatin is available within the electrospun scaffolds after being in contact with an aqueous medium. These results demonstrate that the cross-linking treatment probably prevents the gelatin mass loss in the initial days after a scaffold is implanted as a vascular graft since several physicochemical phenomena associated with biological stages of tissue development occur at different time scales [39]. It has been reported, for example, that cell adhesion develops for hours, while proliferation and differentiation occur on a scale between days and weeks [55, 58]. Therefore, the B1 membrane could have a better performance because it loses a lower mass of gelatin, which would favor cell proliferation and differentiation in the initial days and weeks.

Vascular grafts must ensure adequate properties of mechanical strength and elasticity to mimic the structure of the native blood vessel [26]. The effect of crosslinking did not generate statistically significant differences, possibly because the treatment affects only the gelatin fibers which are found in a smaller proportion than PCL fibers [54]. This synthetic polymer is responsible for giving mechanical integrity to the tubular scaffolds [24].

In this way, it was observed that the scaffolds with the smallest average fiber diameter and average apparent pore area corresponding to those made with the sequential technique had the highest tensile strength [36, 59, 60]. These results indicate that the predominant effect on the mechanical properties of these tubular scaffolds is the morphological characteristics of the PCL because the proportion of gelatin is much lower and because of its low mechanical strength, and the crosslinking treatment is not significant for this property [14]. Besides, intimal hyperplasia may occur due to a compliance mismatch between the graft and native vessel [26, 61], [27] and the elongation effect should be considered as another failure parameter of these tubular scaffolds.

The scaffolds obtained in the present work are within the range of the native vessels compared to the commercial sample analyzed with a diameter of 6 mm, conditions that would possibly avoid generating complications such as distal mechanical trauma, atheromatous or hardening of the blood vessel [1].

Finally, from the cellular behavior B1 has 46 % of apparent pore area greater than C1, which prevents a homogeneous cellularization from being generated on the electrospun scaffold, since the reticulated B1 membrane exhibits two different layers of fibers that are not interrelated with each other, generating a larger pore that prevents cell-cell communication [62]. While, C1 has a smaller pore size, allowing adhesion, proliferation, and generation of fibroblast syncytium. In addition, the crosslinked C1 membranes present a hybrid fibrillar network, which exhibits micrometric-scale fibers that generate large pores that allow cells to freely infiltrate the membrane, and nanometric fibers that facilitate cell adhesion and proliferation [63].

## Conclusions

Our results allowed us to determine the effect of wall morphological characteristics of scaffolds obtained by different electrospinning methods, composed of a synthetic polymer and a protein, on the fluid-mechanical properties evaluated in an *in vitro* model, for their potential use as vascular grafts with a diameter of 6 mm. For this purpose, a synthetic polymer such as PCL was used taking advantage of its mechanical properties, and a protein as bovine gelatin that improves hydrophilicity and increases biocompatibility. However, this protein is rapidly degraded in aqueous environments, for which a crosslinking process with GA was carried out. It was found an increase in the percentage of beta sheets and turns due to the crosslinking treatment and the rapid dissolution of bovine gelatin when interacting with an aqueous medium would be avoided. In the same way, co-electrospun samples presented a lower mass loss of gelatin, which would possibly favor the attachment points with the cells due to the amino acid sequences of the gelatin. Additionally, the average diameter, the alignment of fibers and the apparent pore area in co-electrospun samples had higher values which improve the cell growth along the scaffold.

It was found that the reticulated membranes favor the adhesion and proliferation of 3T3 fibroblasts, which indicates that the type of co-electrospinning techniques generates biocompatible hybrid fibrillar morphologies. Additionally, the co-electrospinning technique with the lower flux of PCL changes the microarchitecture with a morphology that favors anchoring sites for syncytium that with the time, homogeneously cellularize the surface of the scaffold.

Finally, the tubular scaffolds obtained by sequential or co-electrospinning had a better performance as vascular grafts than the commercial sample analyzed for applications with internal diameters of 6 mm, since they have better permeability and wettability, interconnected pores and a circumferential tensile strength that is more similar to that of a native vessel.

## Data Availability

All relevant data are within the paper and its Supporting Information files.
